# Social Inferences From Faces as a Function of the Left-to-Right Movement Continuum

**DOI:** 10.3389/fpsyg.2020.01488

**Published:** 2020-07-06

**Authors:** Rita Mendonça, Margarida V. Garrido, Gün R. Semin

**Affiliations:** ^1^William James Center for Research, ISPA – Instituto Universitário, Lisbon, Portugal; ^2^ISCTE – Instituto Universitário de Lisboa, Centro de Investigação e Intervenção Social, Lisbon, Portugal; ^3^Faculty of Social and Behavioral Sciences, Utrecht University, Utrecht, Netherlands

**Keywords:** face perception, social inferences, head orientation, eye gaze, face database

## Abstract

We examined whether reading and writing habits known to drive agency perception also shape the attribution of other agency-related traits, particularly for faces oriented congruently with script direction (i.e., left-to-right). Participants rated front-oriented, left-oriented and right-oriented faces on 14 dimensions. These ratings were first reduced to two dimensions, which were further confirmed with a new sample: power and social-warmth. Both dimensions were systematically affected by head orientation. Right-oriented faces generated a stronger endorsement of the power dimension (e.g., agency, dominance), and, to a lesser extent, of the social-warmth dimension, relative to the left and frontal-oriented faces. A further interaction between the head orientation of the faces and their gender revealed that front-facing females, relative to front-facing males, were attributed higher social-warmth scores, or communal traits (e.g., valence, warmth). These results carry implications for the representation of people in space particularly in marketing and political contexts. Face stimuli and respective norming data are available at www.osf.io/v5jpd.

## Introduction

The wealth of information carried by faces may appear to pose a formidable processing task. Nevertheless, people have the remarkable ability to perceive, recognize, memorize and judge faces (Sato and Yoshikawa, 2013) fairly accurately in a matter of milliseconds (Willis and Todorov, 2006). For instance, the rapid attribution of traits to facial stimuli correlates well with actual self-judgments of personality (Penton-Voak et al., 2006). Such convergence alone (among other sources of evidence) attests to the potential of studying human faces in psychology.

Because humans are equipped with a specialized neural network for processing face stimuli they are particularly good at attending to eye gaze in faces (Allison et al., 2000; Hoffman and Haxby, 2000; Hooker et al., 2003). Given their biological and social relevance, human faces are detected and recognized faster than those of primates (Simpson et al., 2014). In fact, the white sclera surrounding the iris is distinct among primates and facilitates the perception of where somebody is looking at (Emery, 2000; Kobayashi and Kohshima, 2001). Eye-gaze, whether direct or averted, has been shown to preferentially capture and engage our attention for distinct reasons (Palanica and Itier, 2012). Direct eye-gaze signals readiness for social interaction, provides a medium for non-verbal communication (Csibra and Gergely, 2009) and for the recognition of certain emotional expressions such as anger (Adams and Kleck, 2003). On the other hand, averted gaze is evolutionarily charged as it may indicate changes in the surrounding environment (Anderson et al., 2003) and activates avoidance motivational brain systems (Hietanen et al., 2008). Because it informs the observer about possible environmental threats, averted gaze triggers automatic shifts of visual attention in the gazed-at direction which is assumed to be of interest to the observer (Friesen and Kingstone, 1998; Driver et al., 1999; Hietanen, 1999; Frischen et al., 2007). The lateralized orientation of social attention is particularly prominent when observing rightward facing gazes due to a cultural asymmetry in visual scanning shaped by the reading and writing habits in Western countries (left-to-right, Suitner et al., 2017). These scanning habits ground the direction in which we conceive human movement, or agency.

The current study was designed to further examine how faces gazing to the left and the right capture attention and whether gaze directionality influences different social inferences. The expectation was that rightward faces, which are consistent with the left-to-right script direction of the participants, will be assigned more agency and agentic-related social inferences than the remaining face directionalities. In the following, we present a brief review of a visual scanning bias – “spatial agency bias” (SAB, for a review see Suitner and Maass, 2016), its mechanisms and the main findings that it has generated. Subsequently, we refer to the literature that draws the implications of SAB for social inferences correlated with agency. Finally, we provide an overview of the current research.

Action is represented as unfolding laterally in the direction of how a native language’s script is written and the direction in which we read. This is also correlated with the syntactic order in a sentence – with the agent (subject) preceding the “patient,” namely the “object” of the action (Maass et al., 2014). These overlapping regularities are reinforced through repeated exposure to spatial layouts in everyday life which are coherent with script direction. Consequently, mental representations of human action are envisaged along a trajectory that correlates with the reading and writing direction along with the syntactical structure of the language one is socialized in. Thus, action progresses from left-to-right in languages such as English and French and right-to-left in languages such as Arabic and Hebrew, and the agent of the action typically occupies the left or right position, respectively, in spatial representations (Maass and Russo, 2003; Stroustrup and Wallentin, 2018; Wallentin et al., 2019).

These spatial biases are also known to influence other important aspects of social life such as artwork appreciation as well as perceptions of sport events. For example, Maass et al. (2007) found that Italian participants perceived a goal in football as more beautiful and stronger and a boxing scene as more violent and harmful, when the direction of action was presented as moving from left-to-right rather than the reverse. Interestingly, these results were found to reverse for Arabic speaking participants.

The systematic link between gender stereotyping and spatial imaging was first shown by Chatterjee (2002), Chatterjee et al. (1999). He reported that men are typically portrayed facing right to convey higher agency, a basic dimension stereotypically associated with males (Abele, 2003). Females, however, are predominantly represented facing left. The asymmetrical rightward bias also facilitates gender categorization. Male faces, relative to female faces, are categorized faster when their profile is presented facing right (Suitner et al., 2017). The spatial representation of stereotypically agentic groups (e.g., males, young people) also follows the culturally determined script direction. In Western countries agentic groups are systematically placed to the left of groups with less agentic qualities (e.g., females, old people) (Maass et al., 2009; Abele and Wojciszke, 2014).

Importantly, these horizontal asymmetries have numerous implications for person perception and are likely to shape social judgments (Maass et al., 2007). Notably, when judging someone as agentic, by association, we often endow them with additional qualities such as power, dominance, competitiveness, and ambition (Hitlin and Elder, 2007). Indeed, in different research traditions with different approaches, the same attributes often emerge with converging patterns of results (Fiske et al., 2002; Abele, 2003; Oosterhof and Todorov, 2008).

For example, Fiske et al. (2002) have proposed that group stereotypes are captured by two primary dimensions namely warmth and competence. A similar proposal by Oosterhof and Todorov (2008) suggests that two dimensions account for multiple trait inferences drawn from emotionally neutral faces. These are the valence component, comprising of trait judgments such as attractiveness and responsibility and a dominance component comprising of judgments such as aggressiveness, dominance, and confidence. These dimensions are semantically and functionally convergent with those proposed by Fiske et al. (2002) as well as with other authors before them (e.g., affiliation and dominance, Wiggins, 1979; communion and agency, Bakan, 1996).

Although people rely on numerous traits when evaluating faces, these are correlated with each other and appear to be summed in two fundamental dimensions, which relate to the appraisal of threat (Oosterhof and Todorov, 2008). One dimension is generally informative of others’ positive or negative intent and the other communicates strength and the diligence to pursue these intentions. Agentic-related traits (e.g., active, industrious) are likely to fall into the latter dimension, as they relate to the ability to dynamically implement and achieve one’s goals. Evidently, there are marked differences in the attribution of these two fundamental dimensions across males and females. Men are systematically perceived as more dominant and agentic, whereas women are often endowed with communal-related traits (Abele, 2003).

Despite the evidence pointing to the convergence of dominance and agency-related traits on the same dimension employed for face evaluation, there is, to our knowledge, no study that has directly examined whether the reported bias in agency attributions generalizes to other important social properties. This was the main goal of the current study.

The aim of the study reported here was to examine the types of social inferences that are likely to be shaped by face and gaze orientation (left, frontal, right). To this end, we integrated a range of adjectives as possible inference categories that have been used: (a) in research documenting the spatial agency bias (Maass et al., 2009; Suitner et al., 2017); (b) in research showing the two-dimensional reduction from trait judgments of faces (Oosterhof and Todorov, 2008; Walker and Vetter, 2009); (c) in recent impression formation literature yielding a two-dimensional solution (Fiske et al., 2007) comparable to research on trait judgments of faces; (d) in other face perception studies (Garcia-Marques et al., 2004; Langner et al., 2010; Ma et al., 2015; O’Reilly et al., 2016; Garrido et al., 2017), and finally, (e) in research showing the grounding of abstract categories of time and politics in a horizontal left-to-right dimension (Santiago et al., 2007; Lakens et al., 2011; Farias et al., 2013, 2016).

A careful examination of these diverse but converging literatures led to the selection of 14 trait categories: attractiveness, familiarity, emotion, valence, activity/passivity, strength, speed, trustworthiness, dominance, competence, warmth, agency, temporal and ideological orientation. To examine how social inferences on these categories would be affected as a function of the head orientation we proceeded in three-steps. First, we conducted an exploratory factor analysis (EFA) to determine the minimum number of common factors required to adequately reproduce the fourteen trait categories. In a second step, we performed a confirmatory factor analysis (CFA) on a separate independent sample to establish the reduction of the fourteen categories to a two-factor structure suggested in the EFA. Finally, and to address the main goal of this research, we analyzed how a target person’s face would be rated on the two established dimensions as a function of head orientation (left vs. front vs. right), the target’s gender (male vs. female), as well as the participants’ gender (male vs. female).

We expected the dimension encompassing agency perceptions, along with other traits loading highly on this dimension (e.g., dominance, strength), to be systematically affected by head orientation. Specifically, right-oriented target faces would lead to a stronger endorsement of the agency related dimension relative to the left-oriented target faces, with front orientation taking intermediary values. Moreover, we expected an influence of the target gender on judgments related to this dimension namely that male targets would be judged higher on this dimension than female targets. Consistent with prior literature reporting that two dimensions suffice to capture trait inferences and intentions (threatening or otherwise) from faces of conspecifics, we expected the emergence of a “softer” second dimension typified with categories such as emotion, valence, or trustworthiness. Congruent with a range of earlier findings (Wiggins, 1979; Wojciszke, 1994; Prentice and Carranza, 2002; Cuddy et al., 2009), we anticipated target gender to show systematic effects on this second dimension with female targets obtaining higher scores on communal-related traits.

## Exploratory Factor Analysis

In order to investigate the underlying structure of the set of fourteen trait categories mentioned above, we conducted a preliminary exploratory factor analysis.

### Materials and Methods

Bellow we report how we determined our sample size, all data exclusions (if any), all manipulations, and all measures in the study.

#### Participants

A total of 223 Portuguese speaking participants (166 females; *M*_age_ = 22.11; *SD* = 13.92) recruited through Prolific Academic crowdsourcing platform answered an online survey using Qualtrics Research Suite Software. The sample size was determined based on at least 50 evaluations per target photo (*n* = 43 models). Since each participant evaluated ten randomly selected target photos, the sample size was set to 215. Because data collection was set to stop at the end of the day the sample reached the required number of participants, the sample was slightly larger.

#### Materials

**FIGURE 1:**
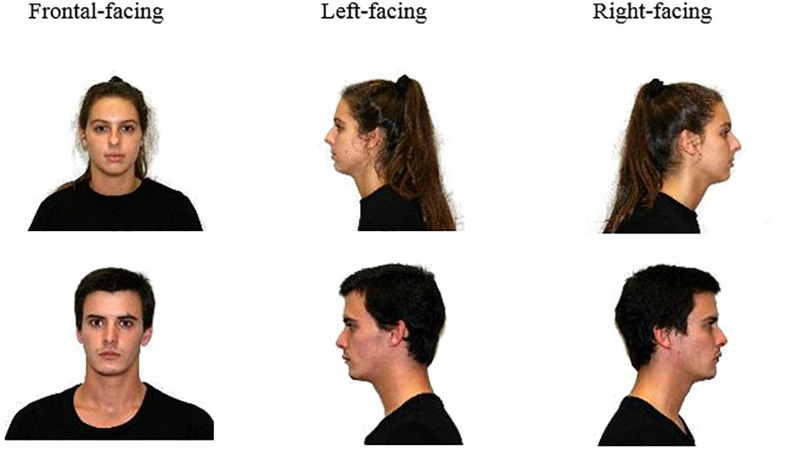
Sample stimuli included in the dataset.

A carefully developed face set comprising 43 models (22 female; *M*_age_ = 20.98, *SD* = 2.26) displaying the three head orientations (left-facing, front-facing, right-facing) (see Figure 1) was used as stimulus materials (for details regarding the development of the stimuli set see Supplementary Material p. 1).

#### Procedure and Measures

The total set of 129 photos (43 models × 3 head orientations) was used in the study. To prevent demotivation and keep the study relatively short, each participant was asked to rate only 10 randomly chosen models from the database. Due to a randomization issue in the survey program, some participants rated less than 10 photos. Nevertheless, each model obtained a minimum of 38 evaluations. A given target model was only presented once in one of the three head orientations to each participant. This manipulation allowed us to rule out possible interference effects such as familiarity with the stimuli.

The study has received full ethics clearance from the Ethics Committee of the host institution. All participants provided informed consent on the first page of the survey and were free to withdraw at any point in time. First, the instructions of the task and a description of the 14 items and scale endpoints were presented. Participants then evaluated a subset of ten random photos on the 14 scales without any time limit. For each photo the respective rating item was shown below the image (e.g., “Dominance”) and the corresponding scale anchors were displayed below it. The same photo appeared until the 14 scales were rated. Then a new photo-scale pair was presented, and so on. Photos and items were randomly presented.

### Dimensions of Interest

Subjective ratings of the 14 dimensions were collected in 7-point scales (see Table 1)^1^.

**TABLE 1:** Scales and endpoints presented in the survey.

Scales	Endpoints	
	1	7
Attractiveness	Very unattractive	Very attractive
Familiarity	Not familiar at all	Very familiar
Emotion	Does not display any emotion	Displays a lot of emotion
Valence	Very negative	Very positive
Activity/Passivity	Very passive	Very active
Strength	Very weak	Very strong
Speed	Very slow	Very fast
Trustworthiness	Not trustworthy at all	Very trustworthy
Dominance	Not dominant at all	Very dominant
Competence	Not competent at all	Very competent
Warmth	Very cold	Very warm
Agency	Not proactive at all	Very proactive
Temporal Orientation	Very past oriented	Very future oriented
Ideological Orientation	Very conservative	Very progressive

### Results

Data were collected for all 129 photos. All participants responded to the entire set of scales in each subset of ten photos leaving no missing data. Additionally, we checked participants’ ratings and found no indication of systematic use of the same value of the 7-point scale, therefore no responses were excluded.

In order to test for participants’ ratings across the 14 scales, we split the total number of responses in two subsamples of similar size (*n_1_* = 949; *n*_2_ = 904) randomly selected from the main sample and found no significant differences between the subsamples, all *t*s < 1.

We started by submitting the 14 scales to an EFA using principal components extraction with direct oblimin rotation. In order to further establish reliability, we conducted the analysis by having the data file randomly split into two halves. In both analyses, a similar two-factor solution emerged (for details see Supplementary Material pp. 1–2; Supplementary Table S1). Consistency of participants’ ratings proved to be reliable and we proceeded with a principal component analysis for the entire dataset, which resulted in a two-factor solution explaining 53.08% of the total variance. Scales of activity/passivity, strength, dominance, agency, speed, temporal orientation, and ideological orientation loaded highly on Factor 1. Scales of attractiveness, familiarity, emotion, valence, trustworthiness, and warmth loaded highly on Factor 2. Interestingly, competence presented a similar contribution to both factors (for details see Supplementary Material p. 3; Supplementary Table S2).

## Confirmatory Factor Analysis

Before assessing the main hypothesis driving this research, we performed a CFA on an independent sample to verify whether the proposed two-factor structure identified in the EFA presented an adequate fit.

### Materials and Methods

#### Participants

We recruited 360 participants (121 females, *M*_age_ = 22, *SD*_age_ = 5.39) through Prolific Academic crowdsourcing platform to participate in the online survey programmed in Qualtrics Research Suite Software. Participants were screened for Portuguese nationality and Portuguese as native language. Sample size was determined based on the following rationale: each participant was assigned to a block of 10 (*n* = 3 blocks) or 11 (*n* = 9 blocks) randomly selected photos from the entire set (*n* = 129) until each block had been rated by 30 participants. Each of the 129 photos was rated in the 14 dimensions by 30 participants.

#### Procedure and Measures

The procedure and dimensions of interest for the online data collection were a replication of those employed in the EFA. Participants gave their informed consent stating that their responses would be anonymous and that they could stop the survey at any time by closing the browser window. Participants rated a set of either 10 or 11 randomly selected photos in distinct face orientations. Notably, we established quotas to ensure that each stimulus photo in the three head orientations was presented to different participants. Thus, a given participant never rated the same model with different head orientations. This means that each model (*n* = 43) was evaluated 90 times in the 14 dimensions, 30 in each face orientation. Photos were presented individually and paired with a given dimension and its scale anchors until all 14 dimensions were presented. After the 14 ratings, a new photo-dimension appeared and so on. Participants had no time constraints to respond to each question, but a time-limit (40 min.) was established to complete the entire survey. Photos and items were randomly presented.

### Results

We conducted a CFA with maximum likelihood estimation using AMOS 26. We did not detect any systematic use of the same scale points and there were no missing responses thus no participant was excluded. Importantly, the competence item had obtained an equivalent contribution to both factors in the EFA. Competence has conventionally been treated in previous two-dimensional models as part of the dominance/power trait judgments (Oosterhof and Todorov, 2008). We therefore included competence in Factor 1 of our proposed model (Model 1, for details see Supplementary Material pp. 3–4, Supplementary Figure S1) but have nevertheless tested an alternative model with the competence item loading on Factor 2 (Model 2, for details see Supplementary Material, p. 5, Supplementary Figure S2). The alternative model rendered poorer adjustment indices and thus we proceeded with the analysis for the Model 1.

Factor loadings in the new sample were smaller than those obtained in the sample used in the EFA. Notwithstanding, we replicated the same dual structure with all items loading above 0.30 (Hair et al., 2014) on the corresponding construct and being statistically significant in the predicted directions (*p* < 0.001). The two-factor model had a model chi-square of 703.387 (d.f. = 72, *p* < 0.001). The model chi-square fit index is very sensitive to sample size and is no longer considered as a basis for acceptance or rejection (Vandenberg, 2006) because “its sensitivity to discrepancies from expected values at increasing sample sizes can be highly problematic if those discrepancies are considered trivial from an explanatory-theory perspective” (Barrett, 2007, p. 815). Considering that we have a large number of observations, we used as further goodness-of-fit indices the Tucker-Lewis index (TLI = 0.90), the Normed Fit Index (NFI = 0.91), the Comparative Fit Index (CFI = 0.91), the Goodness of Fit Index (GFI = 0.97), and the Root Mean Square Error of Approximation (RMSEA = 0.048, *CI* [0.044, 0.051], *p* > 0.250). These values meet the recommended criteria for TLI, NFI, CFI, and GFI greater than 0.90 and RMSEA lower than 0.06 (Hu and Bentler, 1999; Marôco, 2014). Thus, the fit of the model was considered good.

The reliability of the internal scores was assessed through Mcdonald’s omega, a more appropriate reliability coefficient for bifactor models than Cronbach’s alpha namely under the assumption that errors may be correlated (Raykov, 2001). Reliability was satisfactory for the power dimension (ω = 0.73), and below the recommended threshold for the social-warmth dimension (ω = 0.56) although exceeding the suggested minimum of 0.50 (Reise, 2012). To ensure that the individual weight of each item reflected on each latent factor, scores were saved in the original database using the regression method. Factor scores were then standardized and, attending to the nature of each set of traits, correspondingly labeled “power” dimension (Factor 1) and “social-warmth” dimension (Factor 2).

## Subjective Rating Norms

To address the main goal driving this paper, we investigated whether face inferences regarding the two obtained dimensions of power and social-warmth are a function of the head orientation (left vs. front vs. right), the target’s gender (male vs. female), as well as the participants’ gender (male vs. female). To this end, we conducted a multivariate analysis of variance (MANOVA).

Significant multivariate main effects of head orientation [Pillai’s trace = 0.029, *F*(4, 7716) = 28.267, *p* < 0.001, $n_{\text{p}}^{2}$ = 0.014] and of target gender [Pillai’s trace = 0.007, *F*(2, 3857) = 12.637, *p* < 0.001, $n_{\text{p}}^{2}$ = 0.007] were observed. No main effect of participant’s gender was observed (*p* > 0.250). A further interaction effect of head orientation and target gender emerged [Pillai’s trace = 0.003, *F*(4, 7716) = 2.542, *p* = 0.038, $n_{\text{p}}^{2}$ = 0.001].

**FIGURE 2:**
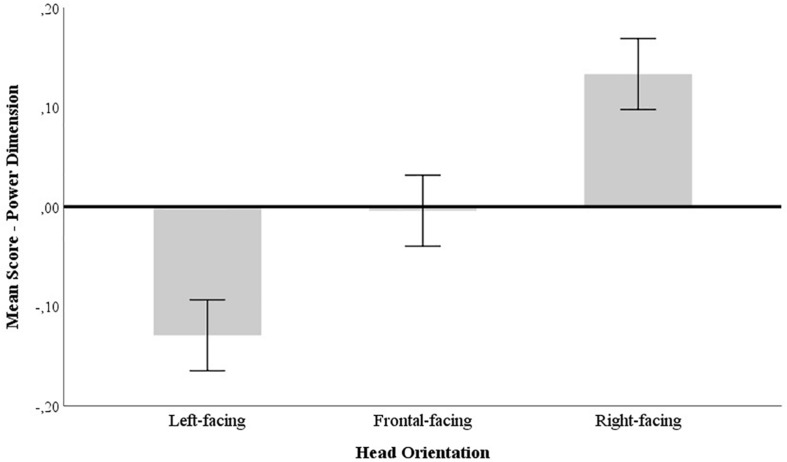
Mean differences for left, front, and right head orientations in the Power scale. Error bars represent the 95% confidence interval.

**FIGURE 3:**
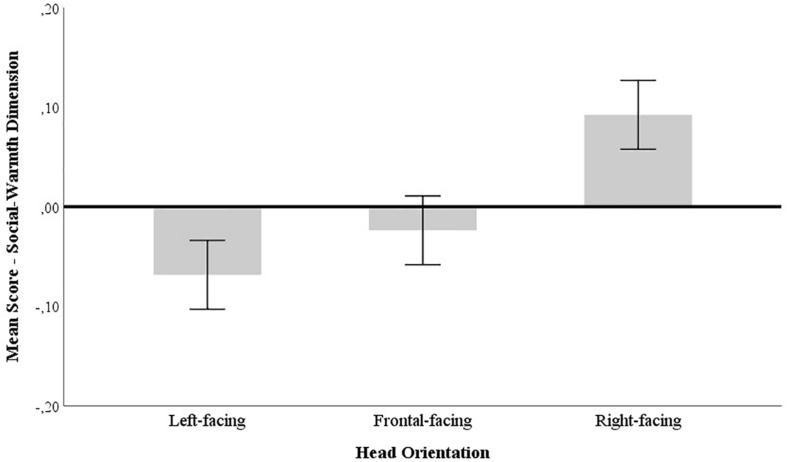
Mean differences for left, front, and right head orientations in the Social-Warmth scale. Error bars represent the 95% confidence interval.

Subsequently, we examined the univariate main effects of head orientation and target gender, and of the interaction between the two. A significant main effect emerged across both power [*F*(2, 3858) = 49.181, *p* < 0.001, $n_{\text{p}}^{2}$ = 0.025] (see Figure 2) and social-warmth dimensions [*F*(2, 3858) = 19.913, *p* < 0.001, $n_{p}^{2}$ = 0.10] (see Figure 3). *Post hoc* comparisons were performed to determine the significance of pairwise contrasts using the Bonferroni correction. In the power dimension, right-facing faces (*M* = 0.132, *SE* = 0.019) obtained significantly higher power scores than front-facing faces (*M* = −0.009, *SE* = 0.019; *p* < 0.001, *CI* [0.077, 0.205]) and left-facing faces, which had the overall lowest power scores (*M* = −0.132, *SE* = 0.019; *p* < 0.001, *CI* [0.200, 0.328]). Faces presented in a frontal perspective also obtained significantly higher power attributions than faces presented in a leftward perspective (*p* < 0.001, *CI* [0.059, 0.187]).

Although the mean differences were less pronounced for social-warmth attributions than for power judgments, social-warmth scores for right-facing faces (*M* = 0.088, *SE* = 0.018) were also significantly higher than for faces of models in frontal (*M* = −0.026, *SE* = 0.018; *p* < 0.001, *CI* [0.053, 0.176]) and left-facing perspectives (*M* = −0.070, *SE* = 0.018; *p* < 0.001, *CI* [0.096, 0.220]). Standardized mean differences in social-warmth related traits were not significant between front-facing and left-facing faces (*p* > 0.250).

As for the effect of target gender, although having yielded a multivariate effect, we did not observe significant differences in the univariate effects on power [*F*(1, 3858) = 2.888, *p* = 0.089, $n_{p}^{2}$ = 0.001] and social-warmth dimensions [*F*(1, 3858) = 1.877, *p* = 0.171, $n_{p}^{2}$ = 0.000]. Finally, the interaction between head orientation and target gender yielded significant differences on the social-warmth ratings [*F*(2, 3858) = 3.755, *p* = 0.023, $n_{p}^{2}$ = 0.002] but not on the power ratings [*F*(2, 3.755) = 0.881, *p* > 0.250, $n_{p}^{2}$ = 0.000]. When presented with front-facing faces, participants rated females with higher social-warmth (*M* = 0.12, *SE* = 0.026) than males (*M* = −0.062, *SE* = 0.025, *p* = 0.037, *CI* [0.05, 0.147]). These findings are not surprising given that the attribution of communal traits to females over males has been repeatedly reported in the literature (Abele, 2003).

In order to better understand how face directionality drives power-related judgments, we examined the individual contribution of the eight items previously found to have higher loadings in the power dimension as a function of the head orientation of the models in a multivariate analysis of variance. Once again head orientation yielded a significant multivariate main effect [Pillai’s trace = 0.046, *F*(16, 7722) = 11.386, *p* < 0.001, $n_{p}^{2}$ = 0.023]. Moreover, statistically significant univariate main effects for head position emerged across all eight items of this dimension: activity/passivity, *F*(2, 3867) = 10.184, *p* < 0.001, $n_{p}^{2}$ = 0.005; strength, *F*(2, 3867) = 15.600, *p* < 0.001, $n_{p}^{2}$ = 0.008; dominance, *F*(2, 3867) = 38.970, *p* < 0.001, $n_{p}^{2}$ = 0.020; competence, *F*(2, 3867) = 5.373, *p* = 0.005, $n_{p}^{2}$ = 0.003; agency, *F*(2, 3867) = 16.508, *p* < 0.001, $n_{p}^{2}$ = 0.008; speed, *F*(2, 3867) = 30.746, *p* < 0.001, $n_{p}^{2}$ = 0.016; temporal orientation,*F*(2, 3867) = 46.582, *p* < 0.001, $n_{p}^{2}$ = 0.024; and ideological orientation, *F*(2, 3867) = 32.492, *p* < 0.001, $n_{p}^{2}$ = 0.017.

A Bonferroni *post hoc* analysis was performed to examine individual mean difference comparisons across head orientations and the eight items loading highly on the power dimension, which previously revealed a significant main effect (see Table 2).

**TABLE 2:** Mean difference comparisons between right-facing, front-facing, and left-facing perspectives in scales of activity/passivity, strength, dominance, competence, agency, speed, temporal orientation, and ideological orientation.

			Mean difference	SE	*p*	95% CI	
						Lower bound	Upper bound
Activity/Passivity	Right-facing	Frontal-facing	−0.139*	0.056	**0.042**	0.004	0.274
	(*M* = 4.02)	Left-facing	−0.254*	0.056	**0.000**	0.119	0.389
	Frontal-facing	Left-facing	0.116	0.056	0.122	–0.020	0.251
	(*M* = 3.88)	Right-facing	−0.139*	0.056	**0.042**	–0.274	–0.004
	Left-facing	Frontal-facing	0.116	0.056	0.122	–0.251	0.020
	(*M* = 3.76)	Right-facing	0.254*	0.056	**0.000**	–0.389	–0.119
Strength	Right-facing	Frontal-facing	0.130*	0.054	**0.047**	0.001	0.259
	(*M* = 4.18)	Left-facing	0.300*	0.054	**0.000**	0.171	0.429
	Frontal-facing	Left-facing	0.170*	0.054	**0.005**	0.041	0.299
	(*M* = 4.05)	Right-facing	−0.130*	0.054	0.047	–0.259	–0.001
	Left-facing	Frontal-facing	−0.170*	0.054	**0.005**	–0.299	–0.041
	(*M* = 3.88)	Right-facing	−0.300*	0.054	**0.000**	–0.429	–0.171
Dominance	Right-facing	Frontal-facing	0.256*	0.056	**0.000**	0.122	0.390
	(*M* = 4.09)	Left-facing	0.494*	0.056	**0.000**	0.360	0.628
	Frontal-facing	Left-facing	0.238*	0.056	**0.000**	0.104	0.372
	(*M* = 3.84)	Right-facing	−0.256*	0.056	**0.000**	–0.390	–0.122
	Left-facing	Frontal-facing	−0.238*	0.056	**0.000**	–0.372	–0.104
	(*M* = 3.60)	Right-facing	−0.494*	0.056	**0.000**	–0.628	–0.360
Competence	Right-facing	Frontal-facing	0.070	0.053	0.560	–0.057	0.196
	(*M* = 4.24)	Left-facing	0.172*	0.053	**0.003**	0.046	0.299
	Frontal-facing	Left-facing	0.102	0.053	0.158	–0.024	0.229
	(*M* = 4.71)	Right-facing	–0.070	0.053	0.560	–0.196	0.057
	Left-facing	Frontal-facing	–0.102	0.053	0.158	–0.229	0.024
	(*M* = 4.07)	Right-facing	−0.172*	0.053	**0.003**	–0.299	–0.046
Agency	Right-facing	Frontal-facing	0.100	0.055	0.204	–0.031	0.231
	(*M* = 4.16)	Left-facing	0.309*	0.055	**0.000**	0.177	0.440
	Frontal-facing	Left-facing	0.209*	0.055	**0.000**	0.077	0.340
	(*M* = 4.06)	Right-facing	–0.100	0.055	0.204	–0.231	0.031
	Left-facing	Frontal-facing	−0.209*	0.055	**0.000**	–0.340	–0.077
	(*M* = 3.85)	Right-facing	−0.309*	0.055	**0.000**	–0.440	–0.177
Speed	Right-facing	Frontal-facing	0.286*	0.054	**0.000**	0.158	0.415
	(*M* = 4.17)	Left-facing	0.410*	0.054	**0.000**	0.282	0.539
	Frontal-facing	Left-facing	0.124	0.054	**0.062**	–0.004	0.252
	(*M* = 3.89)	Right-facing	−0.286*	0.054	**0.000**	–0.415	–0.158
	Left-facing	Frontal-facing	–0.124	0.054	0.062	–0.252	0.004
	(*M* = 3.76)	Right-facing	−0.410*	0.054	**0.000**	–0.539	–0.282
Temporal Orientation	Right-facing	Frontal-facing	0.186*	0.055	**0.002**	0.055	0.317
	(*M* = 4.46)	Left-facing	0.522*	0.055	**0.000**	0.391	0.654
	Frontal-facing	Left-facing	0.336*	0.055	**0.000**	0.205	0.468
	(*M* = 4.27)	Right-facing	−0.186*	0.055	**0.002**	–0.317	–0.055
	Left-facing	Frontal-facing	−0.336*	0.055	**0.000**	–0.468	–0.205
	(*M* = 3.93)	Right-facing	−0.522*	0.055	**0.000**	–0.654	–0.391
Ideological Orientation	Right-facing	Frontal-facing	0.222*	0.055	**0.000**	–0.349	–0.087
	(*M* = 4.32)	Left-facing	0.440*	0.055	**0.000**	–0.571	–0.309
	Frontal-facing	Left-facing	0.218*	0.055	**0.000**	0.087	0.349
	(*M* = 4.10)	Right-facing	−0.222*	0.055	**0.000**	–0.353	–0.092
	Left-facing	Frontal-facing	−0.218*	0.055	**0.000**	0.087	0.349
	(*M* = 3.88)	Right-facing	−0.440*	0.055	**0.000**	–0.353	–0.092

*p < 0.05, Values in bold represent *p*’s < 0.05.

Notably, in all the above-mentioned items, mean ratings were systematically higher for right-facing photos, followed by frontal-facing and left-facing photos. Thus, mean differences between right and left-facing photos consistently presented the highest values on all item ratings, which strongly suggests a substantial impact of the rightward directionality on social perceptions, particularly on power attributions.

An additional multivariate analysis on the remaining six items with greater contribution to the second, social-warmth dimension was also conducted (for detail see Supplementary Material pp. 5–6; Supplementary Table S3).

To further control for dependencies in ratings driven by the variance introduced by the target models’ photos (photo ID), as well as the participants’ interindividual differences (participant ID), two separate linear mixed models were conducted (LMM, one for each dimension). We started by performing a visual inspection of the residual plots that did not reveal any severe violation of the homoscedasticity or normality assumptions. Both LMM’s were conducted including the photo ID and participant ID as clustering factors, the dimension (power or social-warmth) as the dependent variable, and head orientation, target gender, and participant gender as categorical independent variables.

As fixed effects in the model, we considered the head orientation, the target gender, and the participant gender as well as their second and third-order interactions. As random effects, we included random intercepts per participant and per photo. Moreover, the model was estimated using restricted maximum likelihood, and a Satterthwaite approximation of the degrees of freedom was considered (see West, 2009). The LMM analyses were performed using the GAMLj module (Gallucci, 2019) implemented with the jamovi software (The jamovi project, 2019).

### Power Dimension

The LMM analysis (*R^2^_*marginal*_* = 0.03; *R^2^_*conditional*_* = 0.15), revealed a significant main effect of head orientation [*F*(2, 3510.2) = 56.493; *p* < 0.001]. This main effect confirms that power ratings differed significantly across the three head orientations. Replicating what was observed in the multivariate analysis of variance, *post hoc* comparisons with Bonferroni correction revealed that left-facing faces (*M* = −0.129, *SE* = 0.017) gave rise to lower ratings of power than right-facing faces [*M* = 0.133, *SE* = 0.018; *t*(3510) = −10.61, *p* < 0.001]. Frontal-facing models (*M* = −0.004, *SE* = 0.019) also generated lower power scores than did right-facing models [*t*(3509) = −5.88, *p* < 0.001]. Finally, the mean ratings’ difference between left-facing and front-facing models was smaller, but nevertheless significant [*t*(3511) = −4.74, *p* < 0.001].

Notably, the LMM showed no main effect of target gender [*F*(1, 43.5) = 1.634, *p* = 0.208] nor of participant gender [*F*(1, 360.6) = 0.247, *p* = 0.620]. In addition, we observed no interaction between head orientation and target gender [*F*(2, 3571.8) = 0.752, *p* = 0.472], no interaction between head orientation and participant gender [*F*(2, 3531.1) = 0.378, *p* = 0. 686], no interaction between target gender and participant gender [*F*(1, 3514.4) = 0.847, *p* < 0.357], and no interaction between head orientation, target gender, and participant gender [*F*(2, 3577.8) = 2.070, *p* = 0.126].

### Social-Warmth Dimension

The LMM analysis (*R^2^_*marginal*_* = 0.02; *R^2^_*conditional*_* = 0.11) revealed a significant main effect of head orientation [*F*(2, 3518.1) = 22.5364, *p* < 0.001], once again attesting that distinct head orientations drive social-warmth judgments differently. *Post hoc* comparisons with Bonferroni correction procedure showed that left-facing models (*M* = −0.069, *SE* = 0.017) were judged with lower social-warmth traits than right-facing models [*M* = 0.092, *SE* = 0.017; *t*(3518) = −6.43, *p* < 0.001]. Front-facing models (*M* = −0.024, *SE* = 0.018) were also attributed lower social-warmth scores than right-facing models [*t*(3517) = −4.90, *p* < 0.001]. Similarly to what was observed in the multivariate analysis of variance, the attribution of social-warmth judgments was not different across left and frontal face perspectives [*t*(3519) = −1.53, *p* = 375].

We observed an additional interaction effect between head orientation and target gender [*F*(2, 3581.8) = 3.4782, *p* = 0.031]. *Post hoc* comparisons showed that males presented in frontal perspectives (*M* = −0.060 *SE* = 0.025) obtained significantly lower social-warmth scores than males presented in right perspectives [*M* = 0.134, *SE* = 0.024; *t*(3555) = −5.227, *p* < 0.001]. Left-facing males (*M* = −0.100, *SE* = 0.021) were judged lower in social warmth than right-facing females [*M* = 0.048, *SE* = 0.026; *t*(191) = −4.173, *p* < 0.001]. Left-facing males also obtained lower social-warmth ratings than right-facing males [*t*(3556) = −6.099, *p* < 0.001]. Finally, right-facing males obtained higher scores than left-facing female models [*M* = −0.035, *SE* = 0.025; *t*(191) = 3.898, *p* = 0.002]. The remaining pairwise comparisons did not yield significantly different social-warmth ratings (all *p*’s > 0.152).

The LMM revealed no main effect of participant gender [*F*(1, 357.4) = 0.2948, *p* = 0.588], no significant interaction between head orientation and participant gender [*F*(2, 3540.9) = 0.0370, *p* = 0.964], no interaction between participant gender and target gender [*F*(1, 3517.4) = 2.6713, *p* = 102], and no third-order interaction between head orientation, target gender, and participant gender [*F*(2, 3593.6) = 2.9113, *p* = 0.055].

In sum, after entering the photo ID and the participant ID as random coefficients, the systematic effect of rightward faces in power judgments, and to a lesser extent, social-warmth judgments, remained the same. Supplementary Tables S4, S5 (for detail see Supplementary Material, pp. 7–8) provide an overview of the parameter estimates for the main effects and interactions with the aforementioned coefficients in the models.

## Discussion

The main goal of the present study was to investigate whether distinct head orientations drive social inferences differently. Specifically, we speculated that rightward faces, because their directionality overlaps with movement representation in western scripts, would give rise to higher agency and its correlated attributes.

In the current study, we further established that face related judgments can be represented by a two-dimensional factor solution. These dimensions converge with earlier findings (Oosterhof and Todorov, 2008), which identified a valence/trustworthiness dimension and a dominance dimension. Our exploratory analysis yielded a similar two-component solution which was further confirmed by an independent sample: a dimension comprising power-related attributes (e.g., agency, dominance, strength) and a dimension comprising of traits that reflect social-warmth (e.g., familiarity, emotion, attractiveness).

The multivariate analysis of the two dimensions as a function of the three head orientations, target and participant gender yielded the expected main effect for head orientation in the power dimension but also in the social-warmth dimension. Although to a different extent, on both dimensions, we obtained higher scores for right-facing faces relative to frontal and left-facing ones. As hypothesized, this bias was substantially more pronounced on power-related judgments, which seem to be particularly susceptible to head orientation. The power-related ratings were systematically different across all three head orientations, with a clear advantage for right-facing targets, followed by frontal and finally left-facing targets. In addition, after controlling for the potential variance introduced in the ratings by the models’ photos and the individual differences across participants, the results remained the same. This strengthens our account for the systematic impact rightward faces have in driving social inferences, particularly power-related ones.

In examining the univariate effects of the scales with greater weight on the power dimension, we found that aside from the expected agency-related scales (e.g., activity/passivity), the left-right asymmetry characterizes a more general dimension. Right-facing targets also induce attributes of dominance, speed, temporal and ideological orientation significantly stronger than the remaining head orientations. While the specific associations between right-facing orientation and dominance attributions has long been reported (Suitner et al., 2017), the findings on temporal and ideological attributions extend the generality of the head orientation effects.

These findings show that a wide range of social attributes are grounded on a horizontal continuum and are affected by head orientation similarly to what we termed “power” or what was referred to in earlier research as “agency” related attributes. Thus, the left-to-right movement encompasses a generic property that is at the core of how a wide range of categories are grounded. The significance of this work is to be seen in the fact that although these categories are not semantically related, the way they are grounded relies on a unifying principle. We propose that the overlap between agency and a substantial number of distinct but interrelated social categories is a conceptual one. The unifying principle bolstering agency-related properties elicits similar inferences by association, which are sustained by spatial representations flowing congruently with the left-to-right movement. These findings provide a more abstract and integrative framework where agency (Suitner and Maass, 2016), time (Ouellet et al., 2010), and political categories (Mills et al., 2015) are shown to be grounded on a horizontal continuum and can all be primed by head orientation.

It is important to note that the effect of the left-right movement, and by extension that of rightward faces, on social inferences is in all likelihood culture-specific, namely particular to Western script communities like our Portuguese samples (i.e., communities with rightward flowing language script). The opposite preferential representation of agency (i.e., evolving from right-to-left) has been largely reported in leftward flowing languages in distinct attentional and cognitive processes. For instance, line bisection (Chokron and Imbert, 1993), directionality in drawing side view objects (Kebbe and Vinter, 2012), time and number line representation (Dehaene et al., 1993; Ouellet et al., 2010), thematic role drawing tasks (Maass and Russo, 2003), are all heterogeneous but converging examples of how leftward speaking populations preferentially conceived movement as unfolding from right-to-left. However, most reported reversals are considerably weaker in cultures where writing is leftward (Román et al., 2013), likely due to their frequent exposure to westernized spatial layouts whereas exposure to leftward cultures in the West is less frequent. Thus, extrapolations regarding the same pattern of results driven by leftward faces in cultures with right-to-left speaking individuals (i.e., Arabic, Hebrew, Farsi) should be drawn with caution as it is difficult to assess the scope of script directionality effects without a sample from such countries.

The social-warmth dimension revealed that right-facing targets were also judged significantly higher than the left and front-facing targets, which among themselves did not differ. Although smaller in magnitude than on the power dimension, we did not anticipate the effect of head orientation on traits loading on the social-warmth dimension. In fact, previous studies reported faces with direct (relative to averted) gaze as more attractive and trustworthy because they facilitate social communication (Ewing et al., 2010; Kaisler and Leder, 2016). Nevertheless, left-to-right spatial representations seem to facilitate scanning fluency simply because they are script-coherent and hence more familiar (Chae and Hoegg, 2013). Taken together with the script-coherent direction they convey, rightward facing images are also processed with greater ease because they point to an outward direction (Leonhardt et al., 2015). Arguably, this may hint on why we also obtained an advantage, albeit more modest, for right-facing models in social-warmth judgments.

Contrary to what we had hypothesized, we did not observe a main effect of target gender neither on power ratings, particularly for male models, nor on social-warmth judgments, particularly for female models. Although the multivariate main effect of target gender was significant, the univariate effects did not reach statistical significance on either dimension. This pattern, namely the absence of differences on the two dimensions might have resulted from low statistical power. Thus, future studies may require larger samples to uncover potential differences in power and social-warmth traits as a function of the gender of the model. Notably, these results cannot be accounted for by the gender of our participants given that no interaction between these two variables was found.

Although no main effect of target gender was found, a final consideration goes to the interaction between head orientation and target gender, which is in line with our predictions. Models in frontal perspectives were assigned higher social-warmth scores when they were females, relative to males. This result reaffirms the traditional gender roles that emphasize communal-expressive traits in women, conferring them important qualities as nurturing caretakers (Abele, 2003). The effect may be prominent in this south-European sample, where the female gender role is particularly marked. However, this interaction was rather modest (*p* = 0.023). Taken together with the absence of main effect for target gender on social inferences, it may be the case that the features of our specific targets are shaping the results. Therefore, the modesty of the observed gender effects should be interpreted in light of our pool of models, which is composed of young university students who may not fit the imaginary for traditional gender roles and therefore constrain possible effects.

Other authors reporting two fundamental dimensions underlying social perception (Oosterhof and Todorov, 2008) have found the first, primary component capturing face inferences to be the one conveying information on communion-related traits. In contrast, in this study, the power dimension accounted for a higher percentage of the variance in face judgments relative to the social-warmth dimension. Because our participants were not limited to frontal angles but instead produced evaluations on three face perspectives of models, we speculate that rightward oriented faces had a considerable influence on judgments. Arguably, this particular head orientation may have given rise to a substantially higher weight for the power component in overall face evaluation. This means that in a context with multiple face perspectives, power-related traits have the potential to outweigh warmth-related ones and largely contribute to the big picture of face perception.

The results obtained in this study carry important practical and theoretical implications. Research focusing on embodied processes (for reviews see Semin and Smith, 2013; Semin et al., 2013, 2012) and their evaluative consequences could benefit from the manipulation of head orientation and the related norming data made available here (see Supplementary Material for detail). For instance, studies on the embodied categorization of gender frequently rely on faces to assess how abstract dimensions, such as toughness, relate to social categorization (Slepian et al., 2011). Additionally, rightward oriented face stimuli give rise to asymmetries in visual scanning which are likely to affect attentional processes and consequently person perception. Overall, averting the head laterally has been found to modify the perceived social interaction between the observer and the target (Hietanen, 2002). The acknowledgment of the SAB effect on an array of social judgments could also prove useful for practitioners in fields relying on person perception, namely politics (Samochowiec et al., 2010; Farias et al., 2013, 2016), marketing and consumer behavior (Miesler et al., 2010).

These findings constitute a preliminary yet relevant demonstration of how a particular set of social judgments, not necessarily semantically related, are tied to left-to-right distribution in written language. In addition, we build on literature showing that women and men are represented differently in the horizontal vector and capture distinct face inferences (Suitner et al., 2017). Overall, we believe this research highlights the importance of taking the target audience’s script-driven asymmetry into account when representing people in space.

## Data Availability Statement

All datasets generated for this study are included in the article/Supplementary Material and can be found in https://osf.io/v5jpd/.

## Ethics Statement

The studies involving human participants were reviewed and approved by the Ethics Committee, ISPA – Instituto Universitário, Lisboa, Portugal. The patients/participants provided their written informed consent to participate in this study. Written informed consent was obtained from all individuals for the publication of any potentially identifiable images or data included in this article.

## Author Contributions

RM, MG, and GS contributed to the conception and design of the study. RM collected the data, organized the database, and performed the statistical analysis under the supervision of MG and GS. RM and GS wrote the manuscript with input from MG. All authors contributed to the manuscript revision, read and approved the submitted version.

## Conflict of Interest

The authors declare that the research was conducted in the absence of any commercial or financial relationships that could be construed as a potential conflict of interest.

## Footnotes

1 The full stimulus set and respective descriptive data are available on Open Science Framework; www.osf.io/v5jpd.
