# Supplementary material for: Social Inferences From Faces as a Function of the Left-to-Right Movement Continuum
Source: Front Psychol. 2020 Jul 6;11:1488. doi: 10.3389/fpsyg.2020.01488 (PMC7378970; doi:10.3389/fpsyg.2020.01488)
Supplement: Supplementary file 1 [file Table_1.docx]

***Supplementary* *Material***

1. **Development of the Stimuli Set**

Models were university students from the host institution and accepted to participate as volunteers. Photo sessions were conducted individually at the psychology laboratory of the host institution. Upon arrival participants signed an informed consent granting permission for the use of the resulting photos for scientific dissemination purposes. The informed consent clearly stated that the model volunteers would take photos in different perspectives to incorporate a photo database which would be made available for research purposes. To minimize the possibility of interference of extra-facial cues on face judgement, we asked all participants to wear black t-shirts and remove most accessories, glasses, and intense makeup. Nevertheless, we opted to include faces with minor pieces of jewelry, such as earrings, light make-up, and photos of male models with facial hair as we believe these features enhance the ecological validity of the face set.

We used a Canon reflex digital camera (Model EOS 400D) with a resolution of 10.1Mpx and a 40-mm Canon EF-S lens mount. An armchair was positioned against a uniform grey wall and the camera was placed on a tripod fixed at a distance of approximate 90 cm from the chair. The experimenter instructed participants to sit in an upright position and pose with a neutral expression. Photographs were taken from three different perspectives corresponding to three different head positions: frontal, facing left and facing right. Gaze direction was contingent to the head orientation of the models. To ensure minimal display of emotion, several shots were taken per model and per head position so models could practice a neutral expression. For each model and head direction, the photo with overall better quality, position and expression was selected by three independent judges to be included in the final stimuli set. All models in the three head positions resulted in a total of 129 photos (43 for each orientation).

The original dimensions of the photos were 3888 (wide) x 2592 pixels (high). All photographs were then converted from raw to JPEG format, controlled for shadowing, equated for color temperature and the background was corrected for white-balance using Adobe Photoshop CS5 software. An alignment grid was fit over all photos and adjusted accordingly to ensure face position standardization. The resulting photos were cropped and resized into a rectangular frame of 2006 x 2006 pixels to include models’ shoulders and head.

1. **Exploratory Factor Analysis**

As a preliminary first step, the 14 scales were submitted to an exploratory factor analysis. A direct oblimin rotation was employed allowing the factors to remain oblique given that social traits are likely to be correlated. To inspect the reliability of participants’ responses, we conducted a first analysis by having the entire dataset split in two halves. In both analyses, a similar two-factor solution emerged (Table S1), with the first analysis explaining a total of 53.37% of the variance in the first sample and the second analysis explaining a total of 52.97% of the variance in the second sample. Having established the reliability of participants’ answers across the sample, we proceeded with the analysis for the entire dataset from which two factors were extracted and correspondingly labeled (Table S2).

Table S1.

Exploratory factor analysis by random split half with factor loadings for each factor with item loadings above .30.

|  | | |  |  | | |
| --- | --- | --- | --- | --- | --- | --- |
| **Scales** | ***Factor*** | |  | **Scales** | ***Factor*** | |
|  | ***1*** | ***2*** |  |  | ***1*** | ***2*** |
| Attractiveness |  | .505 |  | Attractiveness |  | .564 |
| Familiarity |  | .562 |  | Familiarity |  | .691 |
| Emotion |  | .679 |  | Emotion |  | .628 |
| Valence |  | .596 |  | Valence |  | .523 |
| Activity/Passivity | .764 |  |  | Activity/Passivity | .757 |  |
| Strength | .847 |  |  | Strength | .793 |  |
| Dominance | .867 |  |  | Dominance | .842 |  |
| Trustworthiness |  | .714 |  | Trustworthiness |  | .679 |
| Warmth |  | .894 |  | Warmth |  | .870 |
| Competence | .432 | .313 |  | Competence | .387 | .362 |
| Agency | .643 |  |  | Agency | .688 |  |
| Speed | .734 |  |  | Speed | .738 |  |
| Temporal Orientation | .538 |  |  | Temporal Orientation | .642 |  |
| Ideological Orientation | .423 |  |  | Ideological Orientation | .651 |  |
|  |  |  |  |  |  |  |
| Eigenvalue | 6.218 | 1.254 |  | Eigenvalue | 6.215 | 1.201 |
| Explained Variance | 44.415% | 8.958% |  | Explained Variance | 44.392% | 8.578% |
| Overall Accumulated Variance | 53.373% | |  | Overall Accumulated Variance | 52.970% | |
| KMO and Bartlett’s Sphericity Test | KMO = .931; Chi-Square: 5244.157, p < .001 | |  | KMO and Bartlett’s Sphericity Test | KMO = .930; Chi-Square: 5480.307, p < .001 | |

Table S2.

Exploratory factor analysis to the entire dataset with factor loadings for each factor with item loadings above .30.

| **Scales** | ***Factor 1***  ***(Power)*** | ***Factor 2***  ***(Social-Warmth)*** |
| --- | --- | --- |
| Attractiveness |  | .529 |
| Familiarity |  | .624 |
| Emotion |  | .650 |
| Valence |  | .562 |
| Activity/Passivity | .766 |  |
| Strength | .826 |  |
| Dominance | .864 |  |
| Trustworthiness |  | .708 |
| Warmth |  | .880 |
| Competence | .407 | .338 |
| Agency | .666 |  |
| Speed | .746 |  |
| Temporal Orientation | .592 |  |
| Ideological Orientation | .541 |  |
|  |  |  |
| Eigenvalue | 6.213 | 1.218 |
| Explained Variance | 44.380% | 8.702% |
| Overall Accumulated Variance | 53.082% | |
| KMO and Bartlett’s Sphericity Test | KMO = .935; Chi-Square: 10627.159, p < .001 | |

1. **Confirmatory Factor Analysis**

We submitted the suggested two-factor solution obtained in the EFA to a confirmatory factor analysis with an independent sample. The model was specified with the two latent factors labeled ‘power’ and ‘social-warmth’. The power factor was comprised of the following items: Activity/passivity, strength, dominance, competence, agency, speed, temporal orientation, and ideological orientation. The social-warmth score encompassed the following items: Attractiveness, familiarity, emotion, valence, trustworthiness, and warmth (Model 1, see Figure S1). Furthermore, because the competence trait was found to have a similar contribution to both factors, we tested an alternative model with the item competence on the factor ‘social-warmth’ (Model 2, see Figure S2).

Due to the assessment method (i.e., questionnaire) having similarly worded items, that is, certain items sharing similar content because they are driven from analogous literature, we correlated residuals for items of trustworthiness and warmth, dominance and strength, dominance and activity/passivity, and temporal orientation and ideological orientation (Brown, 2015).


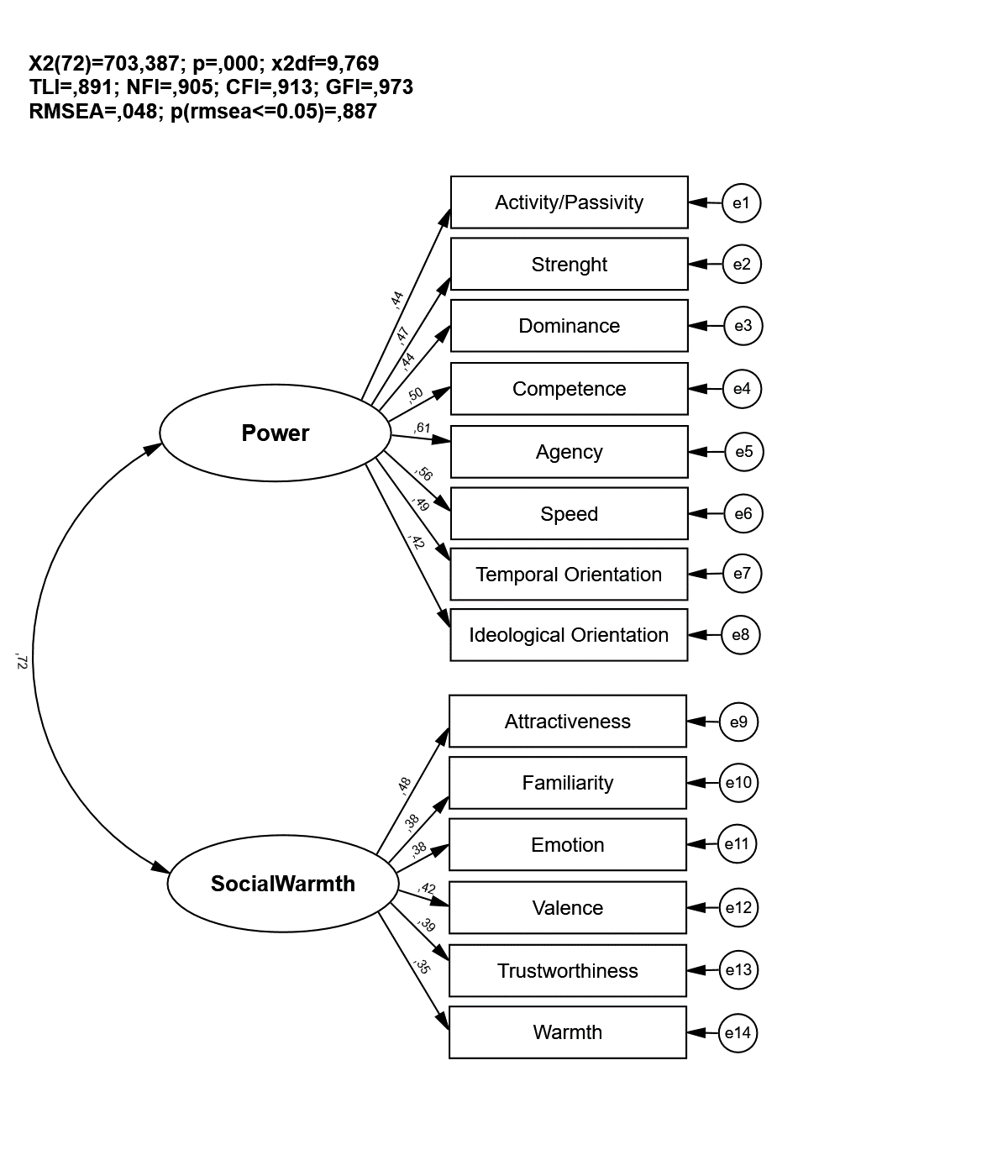


Figure S1. Confirmatory factor analysis (Model 1 – Competence on factor ‘power’)


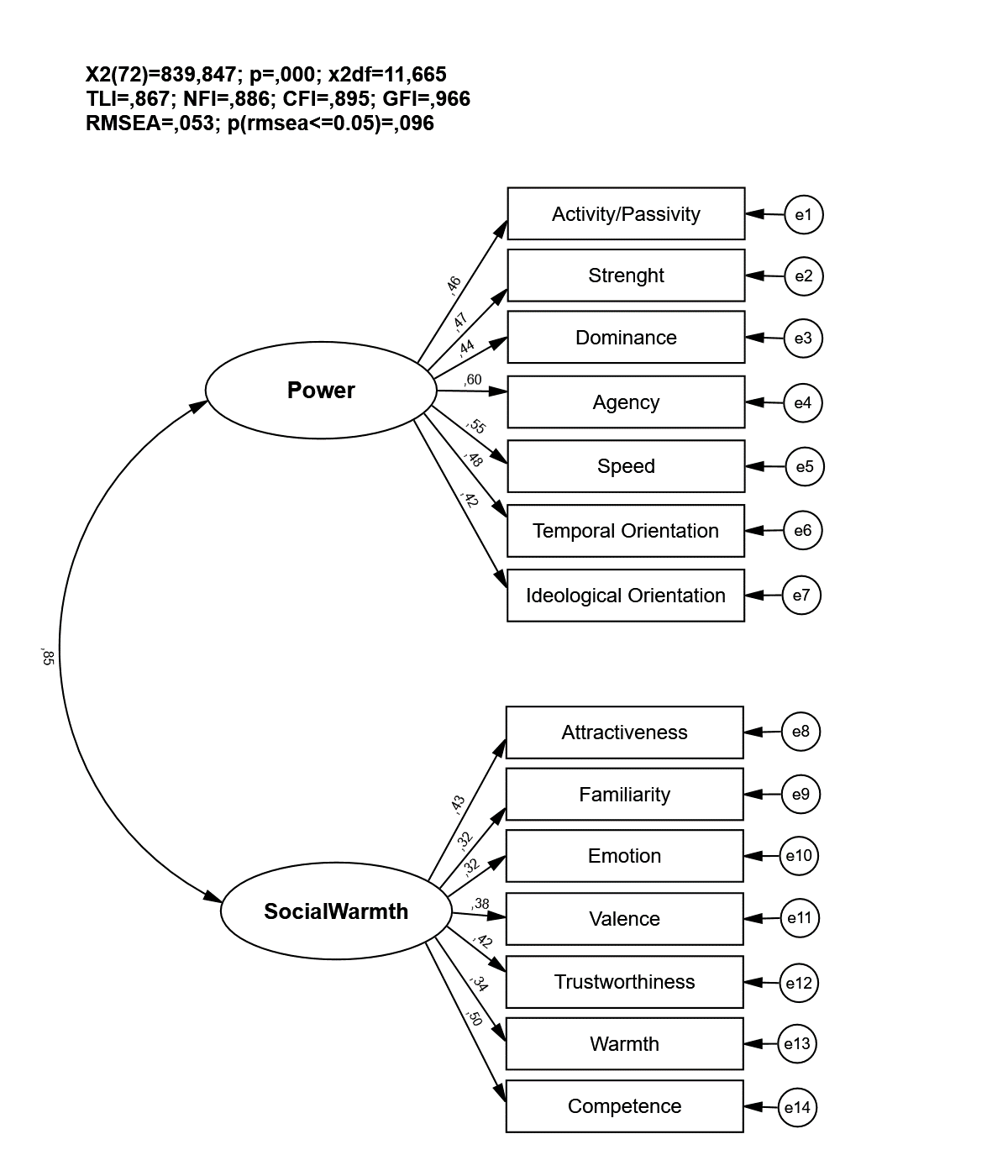


Figure S2. Confirmatory factor analysis (Model 2 – Competence on factor ‘social-warmth’)

**4. Subjective Rating Norms**

As a supplementary analysis we conducted a multivariate analysis of variance to examine the items of attractiveness, familiarity, emotion, valence, trustworthiness, and warmth as a function of the head orientation of the models. The social-warmth dimension, highly driven by the items mentioned above, had been found to be affected by head orientation although to a lesser extent than the power dimension. As expected, head orientation produced a significant multivariate main effect (Pillai’s trace = 0.12, *F*(12, 7726) = 4.041, *p* < .001, $n_{p}^{2}$ = .006). An examination of the univariate main effects revealed that five of the six items were significant, namely attractiveness (*F*(2, 3867) = 3.370, *p* = .034, $n_{p}^{2}$ = .002, familiarity (*F*(2, 3867) =7.229, *p* = .001, $n_{p}^{2}$ = .004), emotion (*F*(2, 3867) = 6.632, *p* = .001, $n_{p}^{2}$ = .003, valence (*F*(2, 3867) = 3.711, *p* = .025, $n_{p}^{2}$ = .002, and warmth (*F*(2, 3867) = 8.283, *p* < .001, $n_{p}^{2}$ = .004). A Bonferroni post-hoc analysis was performed to examine individual mean difference comparisons across the three head orientations and the five items which revealed significant differences (Table S3). Ratings in items of attractiveness, familiarity, and emotion were systematically higher for right-facing faces relative to left-facing faces. Right-facing faces produced higher ratings than front-facing faces in items of valence and warmth.

Table S3. Mean difference comparisons between right-facing, frontal-facing, and left-facing perspectives in scales of attractiveness, familiarity, emotion, valence, and warmth.

|  |  |  | | | **Mean Difference** | **SE** | ***p*** | **95% CI** | |
| --- | --- | --- | --- | --- | --- | --- | --- | --- | --- |
|  |  |  | | |  |  |  | **Lower Bound** | **Upper Bound** |
| Attractiveness |  | Right-facing | | Frontal-facing | .102 | .059 | .243 | -.038 | .243 |
|  |  | (*M* = 3.69) | | Left-facing | .149* | .059 | **.034** | .008 | .289 |
|  |  | Frontal-facing | | Left-facing | .047 | .059 | 1.000 | -.094 | .187 |
|  |  | (*M* = 3.59) | | Right-facing | -.102 | .059 | .243 | -.243 | .038 |
|  |  | Left-facing | | Frontal-facing | -.047 | .059 | 1.000 | -.187 | .094 |
|  |  | (*M* = 3.54) | | Right-facing | -.149* | .059 | **.034** | -.289 | -.008 |
| Familiarity | Right-facing | | Frontal-facing | | .222 | .062 | **.001** | .072 | .371 |
|  | (*M* = 3.79) | | Left-facing | | .184* | .062 | .010 | .034 | .333 |
|  | Frontal-facing | | Left-facing | | -.038 | .062 | 1.000 | -.187 | .111 |
|  | (*M* = 3.56) | | Right-facing | | -.222* | .062 | **.001** | -.371 | -.072 |
|  | Left-facing | | Frontal-facing | | .038 | .062 | 1.000 | -.111 | .187 |
|  | (*M* = 3.59) | | Right-facing | | -.184* | .062 | **.010** | -.333 | -.034 |
| Emotion | Right-facing | | Frontal-facing | | -.010 | .061 | 1.000 | -.157 | .136 |
|  | (*M* = 3.69) | | Left-facing | | .188* | .061 | **.007** | .041 | .334 |
|  | Frontal-facing | | Left-facing | | .198* | .061 | **.004** | .051 | .344 |
|  | (*M* = 3.70) | | Right-facing | | .010 | .061 | 1.000 | -.136 | .157 |
|  | Left-facing | | Frontal-facing | | -.198* | .061 | **.004** | -.344 | -.051 |
|  | (*M* = 3.50) | | Right-facing | | -.188* | .061 | **.007** | -.334 | -.041 |
| Valence | Right-facing | | Frontal-facing | | .136* | .052 | **.028** | .011 | .260 |
|  | (*M* = 4.00) | | Left-facing | | .104 | .052 | .139 | -.021 | .229 |
|  | Frontal-facing | | Left-facing | | -.032 | .052 | 1.000 | -.157 | .093 |
|  | (*M* = 3.86) | | Right-facing | | -.136* | .052 | **.028** | -.260 | -.011 |
|  | Left-facing | | Frontal-facing | | .032 | .052 | 1.000 | -.093 | .157 |
|  | (*M* = 3.90) | | Right-facing | | -.104 | .052 | .139 | -.229 | .021 |
| Warmth | Right-facing | | Frontal-facing | | .216* | .053 | **.000** | .088 | .343 |
|  | (*M* = 3.90) | | Left-facing | | .092 | .053 | .248 | -.035 | .219 |
|  | Frontal-facing | | Left-facing | | -.123 | .053 | .061 | -.251 | .004 |
|  | (*M* = 3.68) | | Right-facing | | -.216* | .053 | **.000** | -.343 | -.088 |
|  | Left-facing | | Frontal-facing | | .123 | .053 | .061 | -.004 | .251 |
|  | (*M* = 3.81) | | Right-facing | | -.092 | .053 | .248 | -.219 | .035 |

As a further analysis, and in order to control for the potential influence of the variance introduced by the photo ID and the participants ID in the subjective ratings, two linear mixed models were conducted (LMM, one for each dimension). Photo ID and participant ID were entered as clustering variables, power and social-warmth dimension as the dependent variables in their corresponding models, and head orientation, target gender, and participant gender as categorical independent variables. Fixed effects were set for variables of head orientation, target gender, and participant gender as well as their 2-way and 3-way interactions. As random effects, we included random intercepts per participant and per photo. Tables S4 and S5 below provide an overview of the estimates for the main effects and interactions with the aforementioned parameters in the models.

Table S4. Principal main effects and interactions, with photo ID and participant ID as random coefficients in the LMM for the power dimension

|  |  |  | ***95% Confidence Interval*** | |  |  |
| --- | --- | --- | --- | --- | --- | --- |
|  | **Effect** | ***B*** | ***Lower*** | ***Upper*** | ***t*** | ***p*** |
| Head Orientation1 | Frontal - Left | .119 | .0697 | .1679 | 4.7432 | < .001 |
| Head Orientation2 | Right – Left | .266 | .2170 | .3153 | 10.6098 | < .001 |
| Target Gender1 | Female - Male | -.039 | -.0991 | .0209 | -1.2784 | .208 |
| Participant Gender1 | Female - Male | -.016 | -.0771 | .0459 | -.4965 | .620 |
| Head Orientation1*Target Gender1 | Frontal – Left * Female-Male | .012 | -.0868 | .1105 | .2359 | .814 |
| Head Orientation2*Target Gender1 | Right – Left * Female - Male | -.047 | -.1455 | .0522 | -.9250 | .355 |
| Head Orientation1*Participant Gender1 | Frontal – Left * Female - Male | -.007 | -.1053 | .0917 | -.1354 | .892 |
| Head Orientation2*Participant Gender1 | Right – Left * Female - Male | .034 | -.0646 | .1326 | .6756 | .499 |
| Target Gender1 * Participant Gender 1 | Female – Male * Female - Male | .038 | -.0426 | .1182 | .9206 | .357 |
| Head Orientation1*Target Gender1*Participant Gender1 | Frontal – Left * Female – Male * Female - Male | -.009 | -.2064 | .1891 | -.0860 | .931 |
| Head Orientation2*Target Gender1*Participant Gender1 | Right – Left * Female – Male * Female – Male | .174 | -.0244 | .3720 | 1.7182 | .086 |

Table S5. Principal main effects and interactions, with photo ID and participant ID as random coefficients in the LMM for the social-warmth dimension

|  |  |  | ***95% Confidence Interval*** | |  |  |
| --- | --- | --- | --- | --- | --- | --- |
|  | **Effect** | ***B*** | ***Lower*** | ***Upper*** | ***t*** | ***P*** |
| Head Orientation1 | Frontal - Left | .038 | -.0105 | -.08595 | 1.535 | .125 |
| Head Orientation2 | Right – Left | .158 | .1101 | .20665 | 6.428 | < .001 |
| Target Gender1 | Female - Male | .027 | -.0256 | .08022 | 1.013 | .317 |
| Participant Gender1 | Female - Male | -.016 | -.0722 | .04090 | -.543 | .588 |
| Head Orientation1*Target Gender1 | Frontal – Left * Female-Male | .012 | -.0844 | .10924 | .252 | .801 |
| Head Orientation2*Target Gender1 | Right – Left * Female - Male | -.106 | -.2034 | -.00937 | -2.149 | .032 |
| Head Orientation1*Participant Gender1 | Frontal – Left * Female - Male | -.007 | -.1039 | .08937 | -.147 | .883 |
| Head Orientation2*Participant Gender1 | Right – Left * Female - Male | .006 | -.0907 | .10295 | .124 | .901 |
| Target Gender1 * Participant Gender 1 | Female – Male * Female - Male | .066 | -.0131 | .14473 | 1.634 | .102 |
| Head Orientation1*Target Gender1*Participant Gender1 | Frontal – Left * Female – Male * Female - Male | .023 | -.1712 | .21683 | .231 | .818 |
| Head Orientation2*Target Gender1*Participant Gender1 | Right – Left * Female – Male * Female – Male | .218 | .0234 | .41237 | 2.196 | .028 |

**References**

Brown, T. (2015). *Confirmatory Factor Analysis for Applied Research, Second Edition*. The Guilford Press.
